# Victor McKusick and the history of medical genetics

**DOI:** 10.1186/2041-2223-4-15

**Published:** 2013-08-01

**Authors:** Kenneth M Weiss

**Affiliations:** 1 Evan Pugh Professor of Anthropology and Genetics, Departments of Anthropology and Biology, Penn State University, University Park, PA 16802, USA

## 

*Victor McKusick and the History of Medical Genetics*. New York: Springer, Krishna R Dronamraju KR, Clair A Francomano CA (Eds); 2012. 232 pages, ISBN 978-1-4614-1677-7.

Over the years, Krishna Dronamraju has been associated in one way or another with some of the leading figures in 20th century genetics. He has produced several books documenting the issues, lives, and work of some of these scientists, perhaps most notably JBS Haldane. The current volume is another such book, which he has edited along with Claire Francomano.

Victor McKusick, who has been called a - if not the - Founding Father of Medical Genetics, was a leading researcher and database developer for the burgeoning young field of human medical genetics. In this manageably small volume, Dronamraju and Francomano have written, assembled and edited reminiscences and thoughtful commentaries on McKusick, both personally and in regard to the issues and his role in advancing them. Some chapters are very short fond memorials, including warm musings by family members including his wife Anne, as well as a variety of obituaries and eulogies to McKusick. These various sections provide vignettes of interactions with him over scientific as well as personal things. In this way, the essentials of McKusick’s biography are covered very readably.

Among his long list of achievements, during his comparably long working life, McKusick did extensive primary research, especially in the Amish, to show the value of isolates and inbreeding to reveal genetic disease. He also provided foundational research on Marfan’s syndrome, dwarfism and numerous other heritable disorders. The chapters in this book provide some details of these topics directly, but also their role in the history of medical genetics and their standing today.

In addition to primary medical genetic research, McKusick organized and ran a leading academic and clinical program in human genetics at the Johns Hopkins medical school. He also led the assembly and maintenance of the catalog, now called Online Mendelian Inheritance in Man (OMIM), to make the rapidly growing, heterogeneous body of knowledge easily available to investigators. This was firstly through several print editions that all of us in human genetics used on a regular basis and then, as the database became unmanageably large and too rapidly changing for print (and web access had become widely available), it morphed into the current online OMIM database. Geneticists still use this on a daily basis.

Also included in this volume’s chapters are miscellaneous photos of McKusick and family or colleagues in various settings and gatherings that give the book a sense of personal history. The editors also provide a bibliography of McKusick’s publications, which spanned 1949 to 2008, an impressive total (even in today’s hasty world) of 772 papers. These papers cover an equally impressive diversity of topics, a testament in itself to his career.

This is not a book about controversies or with many caveats about the approach that McKusick and others took during their active time in human genetics that more recent data have raised, such as the complexity of genetic causation. Only by the later part of his life did heavily scaled up technology make it possible to go much beyond identifying and cataloguing ‘Mendelian’ traits, or exploring the mechanisms behind those that were simple enough to be tractable. But the mid-century human geneticists systematically laid the groundwork for modern human genetics.

In our era of short memory and little interest in conceptual history among current students, McKusick may already be fading as a figure or even a name. We are impatient, and legacy is a luxury that many may feel is not of much interest. However, history does sow the seeds of the present. At least, historians of 20th century genetics will find this a useful collection to consult, a single volume from which to get both a sense of McKusick as a man and as a figure in the history of the science. Unfortunately, for others this will prove to be prohibitively expensive even as an ebook, but it will be one that good libraries will want to have on their shelves.

## Abbreviations

OMIM: Online Mendelian inheritance in man.

## Competing interests

The author declares that he have no competing interests.

